# Expression of the Tobacco Non-symbiotic Class 1 Hemoglobin Gene Hb1 Reduces Cadmium Levels by Modulating Cd Transporter Expression Through Decreasing Nitric Oxide and ROS Level in *Arabidopsis*

**DOI:** 10.3389/fpls.2019.00201

**Published:** 2019-02-22

**Authors:** Ramin Bahmani, DongGwan Kim, JongDuk Na, Seongbin Hwang

**Affiliations:** ^1^Department of Molecular Biology, Sejong University, Seoul, South Korea; ^2^Department of Bioindustry and Bioresource Engineering, Sejong University, Seoul, South Korea; ^3^Plant Engineering Research Institute, Sejong University, Seoul, South Korea

**Keywords:** *ACA10*, cadmium, *CAX*, hemoglobin, *IRT1*, nitric oxide, *PDR8*, ROS

## Abstract

Hemoglobin (Hb) proteins are ubiquitous in plants, and non-symbiotic class 1 hemoglobin (Hb1) is involved in various biotic and abiotic stress responses. Here, the expression of the tobacco (*Nicotiana tabacum*) hemoglobin gene *NtHb1* in *Arabidopsis* (*Arabidopsis thaliana*) showed higher cadmium (Cd) tolerance and lower accumulations of Cd, nitric oxide (NO), and reactive oxygen species (ROS) like hydrogen peroxide (H_2_O_2_). *NtHb1*-expressing *Arabidopsis* exhibited a reduced induction of NO levels in response to Cd, suggesting scavenging of NO by Hb1. In addition, transgenic plants had reduced accumulation of ROS and increased activities of antioxidative enzymes (catalase, superoxide dismutase, and glutathione reductase) in response to Cd. While the expression of the Cd exporters ABC transporter (PDR8) and Ca^2+^/H^+^ exchangers (*CAXs*) was increased, that of the Cd importers iron responsive transporter 1 (*IRT1*) and P-type 2B Ca^2+^ ATPase (*ACA10*) was reduced in response to Cd. When Col-0 plants were treated with the NO donor sodium nitroprusside (SNP) and H_2_O_2_, the expression pattern of Cd transporters (*PDR8*, *CAX3*, *IRT1*, and *ACA10*) was reversed, suggesting that *NtHb1* expression decreased the Cd level by regulating the expression of Cd transporters via decreased NO and ROS. Correspondingly, *NtHb1*-expressing *Arabidopsis* showed increased Cd export. In summary, the expression of *NtHb1* reduces Cd levels by regulating Cd transporter expression via decreased NO and ROS levels in *Arabidopsis*.

## Introduction

Cadmium is a non-essential metal and a major hazardous environmental pollutant because it has toxic effects even at low concentrations. Cadmium negatively influences several aspects of plant metabolism and development, such as growth, transpiration, photosynthesis, respiration, and nutrient distribution (Das et al., 1997; Sanita et al., 1999; Zanella et al., 2016; Ronzan et al., 2017). Moreover, Cd toxicity causes various alterations in plants at genetic, biochemical, and physiological levels and results in phytotoxicity (Wahid et al., 2010). Due to similar oxidation states, Cd can be exchanged with Fe^2+^, Zn^2+^, and Ca^2+^ in some protein structures, leading to protein malfunction (Goyer, 1997). In addition, at excess concentrations, Cd induces the formation of ROS, leading to lipid peroxidation and DNA damage, as well as calcium homeostasis modification (Stohs and Bagchi, 1995). The mechanism of Cd uptake by plants is significantly affected by various factors, such as pH, temperature, aeration, Cd concentration in the environment, and concentration of other micro and macro elements (McLaughlin et al., 1996). Uptake of Cd by plant roots occurs through divalent cation transport systems, including those for iron, zinc, and calcium (Verbruggen et al., 2009).

Several Cd transporters involved in the uptake, efflux, and sequestration of Cd have been identified. Among these proteins, plasma membrane-localized transporters, such as *AtIRT1* (Connolly et al., 2002), *OsIRT1* (Lee and An, 2009), *AhIRT1* (Chen C. et al., 2017), *AtNRAMP1* (Thomine et al., 2000), *AhNRAMP1* (Chen C. et al., 2017), *NtNRAMP1* (Sano et al., 2012), *OsNRAMP5* (Sasaki et al., 2012), *HvNramp5* (Wu et al., 2016), *OsMTP1* (Yuan et al., 2012), *OsZIP1* (Ramesh et al., 2003), and *SaNramp6* (Chen S. S. et al., 2017), have been implicated in Cd uptake. Furthermore, members of the HMA family, including *AtHMA2* (Mills et al., 2003), *AtHMA4* (Hussain et al., 2004; Verret et al., 2004), and *OsHMA2* (Takahashi et al., 2012), as well as the ATP-binding cassette transporter *AtPDR8* (Kim et al., 2007), are located at the plasma membrane and are involved in Cd efflux. In addition, vacuolar transporters, including *AtCAX1* (Wu et al., 2011), *AtCAX2* (Hirschi et al., 2000), *AtCAX4* (Korenkov et al., 2007), *AtHMA3* (Morel et al., 2009), *TcHMA3* (Ueno et al., 2011), *FlHMA3* (Guo et al., 2017), *AtABCC1/AtABCC2* (Park et al., 2012), and *AtABCC3* (Brunetti et al., 2015), play considerable roles in Cd transport into the vacuole.

Calcium (Ca^2+^) is a vital nutrient and signaling molecule that is implicated in various metabolic and signal transduction pathways (Yang and Poovaiah, 2003; Demidchik et al., 2018). It has been reported that Ca interferes with Cd uptake and transport by regulating Cd transporter expressions (Kim et al., 2002; Zeng et al., 2017). Moreover, due to their similar ionic radii, Ca and Cd may compete with each other for uptake and transport into plant cells. It was shown that Cd treatment repressed the activity of Ca^2+^ channels (Li S. et al., 2012). So far, several genes have been found to be involved in Ca^2+^ transport as well as Cd tolerance. Over-expression of *OsACA6* encoding P-type 2B Ca^2+^ ATPase improved Cd tolerance by mediating Cd distribution (enhanced and decreased Cd levels in roots and shoots, respectively) and lowering oxidative stress in tobacco (Shukla et al., 2014). In addition, plant cadmium resistance 1 (PCR1) protein is able to transport Ca^2+^ (Song et al., 2011) and promotes Cd tolerance by reducing Cd accumulation in yeasts and *Arabidopsis* protoplasts (Song et al., 2004).

Nitric oxide is a short-lived free-radical reactive gas that functions in a wide range of physiological processes in plants, such as growth and development, iron homeostasis, and responses to biotic and abiotic stresses (Ramirez et al., 2011). Production of NO in plants is affected by biotic and abiotic stresses (Lamattina et al., 2003; Leitner et al., 2009). NO production in plants under Cd stress conditions can act as either an enhancer or reducer of Cd toxicity. Cd-induced NO production primarily contributes to Cd toxicity through elevating oxidative stress by enhancing ROS, RNS and lipid peroxidation, as well as by repressing the activity of antioxidant enzymes (Groppa et al., 2008; De Michele et al., 2009; Arasimowicz-Jelonek et al., 2012; Kulik et al., 2012). By contrast, NO protects plants against Cd-induced oxidative stress by enhancing antioxidant enzyme activity and reducing ROS accumulation (Verma et al., 2013; Perez-Chaca et al., 2014; Yang et al., 2016). Therefore, a decrease in NO by Cd results in higher ROS levels and toxicity (Rodriguez-Serrano et al., 2009; Ortega-Galisteo et al., 2012; Gupta et al., 2017). Moreover, treatment with exogenous NO donors, such as SNP, mitigates Cd-induced oxidative stress by enhancing the activities of antioxidant enzymes (Kopyra and Gwozdz, 2003; Hsu and Kao, 2004; Li L. et al., 2012; He et al., 2014). In addition, NO accumulation is involved in programmed cell death under Cd toxicity conditions (Ye et al., 2013). NO can also mediate the induction or inhibition of Cd toxicity by increasing Cd accumulation (Ma et al., 2010; Arasimowicz-Jelonek et al., 2012; Chmielowska-Bąk and Deckert, 2013; Han et al., 2014), potentially by enhancing Cd uptake (Besson-Bard et al., 2009; Luo et al., 2012; Zhu et al., 2012) or by decreasing Cd accumulation (Li L. et al., 2012; Zhang et al., 2012; He et al., 2014).

Hemoglobin (Hb), an ubiquitous protein in plants, was first identified in the root nodules of soybean (*Glycine max*) plants and implicated in oxygen binding and transport (Appleby, 1992). There are two classes of plant non-symbiotic *Hb* genes (class 1 and 2), which have over 50% sequence identity, but are distinct in terms of their phylogenetic characteristics, gene expression, and oxygen binding features (Kundu et al., 2003; Igamberdiev et al., 2011). Several lines of evidence indicate a significant role for non-symbiotic class 1 hemoglobin (Hb1) in NO detoxification (Dordas et al., 2003; Igamberdiev et al., 2004; Perazzolli et al., 2004; Hebelstrup et al., 2006). Therefore, Hb1 can participate in plant responses to biotic and abiotic stress by modulating the level of NO. Transgenic *Arabidopsis* plants that overexpress *AtHB1* have higher tolerance to hypoxia stress (Hunt et al., 2002). In addition, tolerance to submergence, salinity, and osmotic stresses are increased by *ZmHb* expression in tobacco (Zhao et al., 2008). Furthermore, heterologous expression of *MsHb1* in tobacco and *Arabidopsis* (Seregelyes et al., 2004; Maassen and Hennig, 2011), *GhHb1* in *Arabidopsis* (Qu et al., 2006), and *AtHb1* in barley (Hebelstrup et al., 2014) improves defense responses against pathogen attack. Down-regulation of cold-responsive genes, as well as mitigated oxidative stress, has also been observed in transgenic *Arabidopsis* expressing *AtHb1* (Cantrel et al., 2011; Thiel et al., 2011). Previous studies have revealed that the growth responses and development of plants are mediated by Hb1, as its expression delays bolting in *Arabidopsis* (Hebelstrup and Jensen, 2008) and reduces plant growth and development in barley (Hebelstrup et al., 2014). Recently, we also showed that over-expression of *NtHb1* enhances Cd tolerance by reducing the NO and Cd levels in transgenic tobacco (Lee and Hwang, 2015). Interestingly, over-expression of spinach *SoHb* in *Arabidopsis* reduces the NO content and tolerance to nitrate, NaCl, and osmotic stresses (Bai et al., 2016). Moreover, *AtHb1*-overexpressing *Arabidopsis* plants fumigated with NO showed improved growth parameters and NO fixation ability compared with the WT plants (Kuruthukulangarakoola et al., 2017). In this study, to understand a mechanism for *Hb1*-induced Cd tolerance in *Arabidopsis*, we over-expressed *NtHb1* in *Arabidopsis* and examined concentrations of Cd, NO, and ROS and expression levels of the Cd transporters Ca^2+^/H^+^ exchangers (*CAXs*), ABC transporter (*PDR8*), and iron responsive transporter 1 (*IRT1*).

## Materials and Methods

### Plasmid Construction and Transformation of *Arabidopsis*

The coding sequence of *NtHb1* (GenBank Access No. KJ808726.1) was amplified by RT-PCR using gene specific primers flanked by XbaI and BamHI restriction sites and sub-cloned into the pBS vector. After sequencing the insert, the XbaI-BamHI fragment was cloned into the binary vector *pBI121*. The *pBI121* vector harboring *NtHb1* was transformed into *Agrobacterium tumefaciens* strain GV3103 by the freeze–thaw method, and plant transformation was achieved by the floral dipping method (Clough and Bent, 1998). To generate *pAtHb1::GUS*-expressing *Arabidopsis*, the promoter region of *AtHb1* (1-kb upstream of the start codon) was amplified using primers with HindIII and XbaI restriction sites, sequenced, and cloned into the binary vector *pBI121*. The primer pairs used for these constructs are shown in Supplementary Table S1.

### Quantitative RT-PCR (Real-Time PCR)

Total RNA was isolated from transgenic *Arabidopsis* seedlings (T_3_ homozygous) grown on 1/2 MS agar plates for 3 weeks using the Plant RNA extraction Kit (Intron, South Korea). After quantification of the RNA using a NanoDrop (BioSpec-nano, Shimadzu, Japan), first-strand cDNA was synthesized from 2 μg of RNA in RNase-free water using PrimeScript^TM^ RT reagent kit (Takara, Otsu, Japan) according to the manufacturer’s instructions. Semi-quantitative PCR was employed for the analysis of *NtHb1* expression by using gene-specific primers. Actin was used as an internal control. The expression levels of cadmium transporter genes in *Arabidopsis* were evaluated by using qRT-PCR. Isolation of total RNA was performed by using transgenic *Arabidopsis* seedlings (T_3_ homozygous) grown on 1/2 MS agar plates supplemented with 0 and 50 μM CdSO_4_ for 3 weeks, and cDNA was synthesized as described above. Quantitative RT-PCR was accomplished using a CFX Connect^TM^ Real-Time PCR Detection System (Bio-Rad Laboratories, Inc., Hercules, CA, United States). The reaction mixture consisted of 10 μl of SYBER Supermix (SsoAdvanced^®^Universal SYBR^TM^ Green Supermix), 1 μl of cDNA, 7 μl of nuclease-free water, and 1 μl of each primer to a final volume of 20 μl. The following qRT-PCR reaction thermal conditions were used: 95°C for 30 s, followed by 40 cycles at 95°C for 15 s and 57°C for 20 s. The *Arabidopsis ACTIN2* gene was used as an internal quantitative control, and the relative expression level of each gene was calculated based on the 2^−ΔΔC^_t_ method (Pfaffl, 2001). Each qRT-PCR experiment was performed three times with different cDNA sets obtained from three independent biological replicates. The gene-specific primers are shown in Supplementary Table S1.

### Western Blot Analysis

*Arabidopsis* wild-type plants (Col-0) were grown on 1/2 MS agar plates containing 0 and 50 μM CdSO_4_ for 3 weeks, and subsequently, the roots and shoots were separated and homogenized in extraction buffer (20 mM Tris–Hcl, pH 8.0, 1 mM DTT (dithiothreitol), 0.3 mM EDTA and protease inhibitor cocktail). Protein samples (40 μg) were loaded and separated on SDS-PAGE gels and then transferred to nitrocellulose membranes (Hybond-C Extra, Amersham Biosciences) and probed with *AtHb1* primary antibodies (Abmart Co.,) and the binding of secondary antibody rabbit anti-mouse horseradish peroxidase-conjugated IgG (Amersham Biosciences) was detected by enhanced chemiluminescence (Amersham Biosciences). Rubisco Ponceau S-stained large subunit was used as a loading control. Three independent biological experiments were performed.

### Analysis of Cadmium Tolerance

To measure cadmium tolerance, seeds of transgenic *Arabidopsis* lines (T_3_ homozygous) were surface-sterilized, germinated and incubated on 1/2 MS agar supplemented with 0 and 50 μM of cadmium sulfate for 3 weeks. This cadmium concentration reduced the fresh weight of wild-type seedlings by 50%. The *Arabidopsis* seedlings were photographed after 3 weeks. These experiments were performed in three independent biological replicates for each line. To calculate the cadmium tolerance, the fresh weight of each seedling (*n* = 90) on cadmium plates was divided by the fresh weight of seedlings grown on control plates (1/2 MS), and finally expressed as the relative fresh weight (%).

### Measurement of Cadmium Concentration in Plants

For the Cd concentration measurement, control and transgenic *Arabidopsis* seeds (T_3_ homozygous) were surface-sterilized, germinated and incubated on 1/2 MS agar supplemented with 50 μM of cadmium sulfate for 3 weeks. The seedlings on each plate were harvested after 21 days, washed with ice-cold 5 mM CaCl_2_ (three times) and dried for 72 h at 60 °C. Then, the dried sample (0.5 g) was digested by using concentrated HNO_3_ and HClO_4_ in a Teflon Digestion Vessel (Savillex, United States). ICP Mass Spectroscopy (Varian 720-MS, United States) was employed to specify the concentration of Cd at a wavelength of 214.44 nm at the National Instrumentation Center for Environmental Management (NICEM) at Seoul National University, South Korea. Three independent biological experiments were performed.

### Localization of Cadmium in Plants

Leadmium^TM^ Green AM (Invitrogen, United States) was used to monitor the localization of Cd in *Arabidopsis* roots following the manufacturer’s instructions. Briefly, control and transgenic *Arabidopsis* seeds were germinated and grown on 1/2 MS medium containing 0 and 50 μM of cadmium. After 21 days, the root samples were harvested and rinsed in distilled water. The working solution of Leadmium^TM^ Green AM was obtained through dilution (1:10) of its stock solution [1 μg/μl in DMSO in 0.85% NaCl. Next, the samples were immersed in staining solution [0.04% (v/v) of Leadmium^TM^ Green AM in 0.85% NaCl] and kept in the dark. After 1 h, the samples were briefly washed with saline solution (0.85% NaCl) (Du et al., 2015) and visualized using a fluorescence microscope (Leica, MZ10F, Germany) with 490 nm excitation and 510–520 nm emission filters under proper magnification (12.5-25X). At least 10 plants were examined per line, and three independent biological experiments were performed.

### ROS (Hydrogen Peroxide) Detection in Roots Using a Fluorescent Probe (CM-H_2_DCFDA)

Visualization of ROS in root was performed by using the CM-H_2_DCFDA [5-(and-6)-chloromethyl-2′,7′-dichlorodihydrofluorescein diacetate] fluorescent probe (Molecular Probes^1^) as previously described (Wang et al., 2014), with some modifications. The roots of three-week-old plants grown in 1/2 MS medium supplemented with 0 and 50 μM of cadmium were harvested and briefly washed with deionized water. After immersing the roots in CM-H_2_DCFDA solution (10 μM) for 15 min in the dark, samples were rinsed with water and photographed using a fluorescence microscope (Leica, MZ10F, Germany) with 485 nm excitation and 510–530 nm emission filters under proper magnification (12.5-20X). Image J software (NIH, United States) was employed for quantification of the fluorescence signal. At least 10 plants were examined per line, and three independent biological experiments were performed.

### Visualization of NO Production in Roots

The NO-specific fluorescent probe DAF-2DA (Sigma Co.) was used to detect NO production as previously described (Besson-Bard et al., 2009), with some modifications. Briefly, the roots of three-week-old plants grown in 1/2 MS medium supplemented with 0 and 50 μM of cadmium were incubated with 10 mM of DAF-2DA (prepared in 20 mM HEPES-NaOH, pH 7.5) under dark conditions for 1 h. Subsequently, the samples were washed with the same buffer and photographed using a fluorescence microscope (Zeiss Axioplan2) with 488 nm excitation and 515–565 nm emission filters. For treatment of NO donor SNP (sodium prusside, Sigma Co.) and NO scavenger cPTIO (Sigma), 500 μM of each chemical was prepared in 20 mM of HEPES-NaOH, pH 7.5 and used for the pre-treatment of the root samples prior to staining with DAF-2DA and visualization. Image J software (NIH, United States) was used for Quantification of the NO signal intensity. At least 10 plants were examined per line, and the experiment was repeated independently three times.

### Measurement of Anti-oxidative Enzyme Activity

Antioxidant enzymes assays were performed using the crude extract of the leaves as the enzyme source. Briefly, leaf samples (0.2 g) of 3-week-old transgenic *Arabidopsis* plants grown on 1/2 MS medium supplemented with or without 50 μM of cadmium sulfate were ground to a fine powder in liquid nitrogen and then homogenized in 1.2 ml of 0.2 M potassium phosphate buffer (pH 7.8 containing 0.1 mM EDTA) and centrifuged at 15,000 × g at 4°C for 20 min to obtain a crude enzyme extract.

Catalase (1.11.1.6) activity, was measured by monitoring the decrease in absorbance at 240 nm as previously described (Aebi, 1984). To start the reaction, leaf crude extract was added to 2.5 μl of H_2_O_2_ (30% solution) prepared in 50 mM of potassium phosphate buffer (pH 7.5) at a final volume of 1 ml. The enzyme activity was calculated according to the extinction coefficient of H_2_O_2_ (40 M^−1^ cm^−1^ at 240 nm) and expressed in terms of millimoles of H_2_O_2_ per minute per gram fresh weight.

To determine the SOD activity (SOD; EC 1.15.1.1), the modified NBT method of Bayer and Fridovich (Beyer and Fridovich, 1987) was employed. Briefly, the assay solution consisted of 50 mM phosphate buffer (pH 7.8) containing 2 mM EDTA, 9.9 mM L-methionine, 55 μM NBT, 0.025% Triton X-100 and 1 mM riboflavin. Following the addition of the leaf extract, the reaction was initiated after exposing the samples to a fluorescent lamp (15 W) for 10 min. The absorbance of the reaction mixture was measured at 560 nm against a similar mixture lacking the leaf extract as a control. The SOD standard curve was used to evaluate the enzyme activity (per gram fresh weight) of the samples.

Glutathione reductase activity (GR; EC 1.8.1.7) was measured as previously described (Smith et al., 1918). The assay solution (1 ml) was prepared in 100 mM potassium phosphate buffer (pH 7.5) and 0.75 mM DTNB, 0.1 mM NADPH, 1 mM GSSG (glutathione disulfide), and 10 μl of leaf extract. GSSG was added to start the reaction, and the absorbance increment at 412 nm was measured when DTNB was reduced to TNB by GSH in the reaction. The activity of GR was determined based on the extinction coefficient of TNB (14.15 M^−1^ cm^−1^) and expressed in terms of millimoles of TNB minute per gram fresh weight. Three independent biological experiments were performed, in which each measurement was repeated three times.

### Measurement of Cd Efflux

Seeds of control (*pBI121*) and *NtHb1*-expressing *Arabidopsis* were germinated and grown on 1/2 MS media plate for 3 weeks, and subsequently, all plants were transferred to 50 μM Cd (cadmium sulfate) agar plates, incubated for 24 h, washed with distilled water, and placed in a Cd-free liquid medium (DW). After 6 and 24 h, the Cd concentrations in the liquid media were determined as described in the above section. Three independent biological experiments were performed.

### *GUS* Staining Analysis

Detection of *AtHb1::GUS* activity was performed as previously described (Prasinos et al., 2005). Briefly, 7-day-old seedlings grown vertically on 1/2 MS agar medium containing 0 and 50 μM of cadmium were incubated in GUS staining solution (50 mM NaPO4 buffer, pH 6.8, 0.5 M EDTA, pH 8.0, 0.5 mM potassium ferrocyanide, 0.5 mM potassium ferricyanide, 20% Triton X-100 and 2 mM X-gluc), for 2 h at 37°C. For short-term experiments, 7-day-old seedlings grown vertically on 1/2 MS agar medium were treated with 50 μM of cadmium for 2, 6, 12, and 24 h. To observe GUS expression in the leaves, the samples were washed with ethanol (70%) to remove green chlorophyll pigments. Images were captured with a microscope (Zeiss Axioplan2). This experiment was repeated 3 times, and 10 seedlings were observed each time.

### Statistical Analysis

All data were subjected to analysis of variance (Two-way ANOVA) by using SAS software (version 9.1), and comparisons of the means were performed according to Tukey’s test at *P* ≤ 0.05 or *P* ≤ 0.01.

## Results

### Over-Expression of *NtHb1* Enhances Cd Tolerance but Decreases Cd Accumulation in Transgenic *Arabidopsis*

**FIGURE 1:**
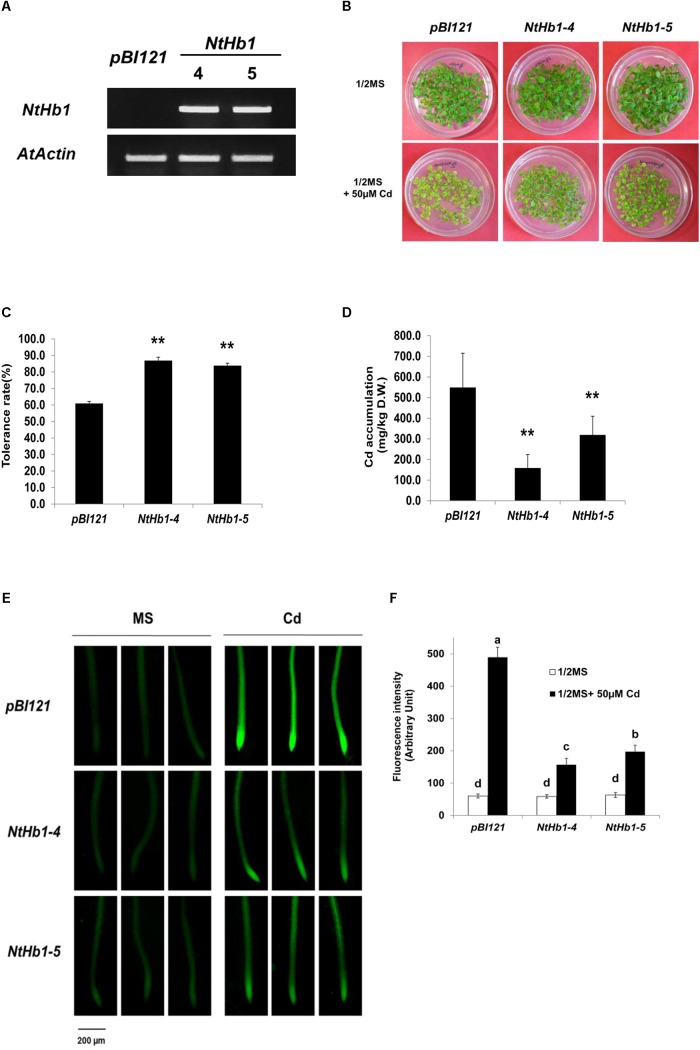
Cd tolerance and accumulation in the control and *NtHb1*-expressing *Arabidopsis*. **(A)** qRT-PCR results showing higher levels of *NtHb1* transcripts in transgenic *Arabidopsis*. Control plants contain the empty vector *pBI121*, and *NtHb1*-4 and *NtHb1*-5 refer to two different lines of *NtHb1*-expressing transgenic *Arabidopsis*. EtBr-stained qRT-PCR product of the actin gene (*AtActin*) was used as a loading control. **(B)** Cadmium tolerance of transgenic *Arabidopsis* expressing *pBI121* (control) and *NtHb1* (*NtHb1*-4 and *NtHb1*-5). Plants were germinated and grown for 3 weeks on 1/2 MS agar plates without (upper) and with (lower) 50 μM CdSO_4_ and then photographed. **(C)** Graph showing the Cd tolerance rates of transgenic *Arabidopsis* shown in **(B)**. Each value corresponds to the means ± standard error (SE) (*n* = 3). **(D)** Accumulation level of Cd in transgenic *Arabidopsis*. Values correspond to the means ± SE (*n* = 3). **(E)** Visualization of Cd in *Arabidopsis* roots using Leadmium^TM^ Green AM. Plants were germinated and grown for 3 weeks on 1/2 MS plates without (left) and with (right) 50 μM Cd. The experiments were performed 3 times, and 10 plants were examined per line for each experiment. **(F)** The intensity of fluorescence (five plants from each line) was measured using Image J software (NIH, United States). Each value corresponds to the means ± SE (*n* = 5). Asterisks indicate significant differences between control and transgenic *Arabidopsis* (*p* ≤ 0.01). Different letters over the bars indicate significant differences according to Tukey’s multiple comparison test (*p* ≤ 0.05).

To examine whether *NtHb1* increases Cd tolerance in *Arabidopsis* similar to that in tobacco (Lee and Hwang, 2015), *NtHb1* was over-expressed in *Arabidopsis* using the binary *pBI121* vector. As shown in Figure 1A, two lines (4 and 5) of transgenic *Arabidopsis* expressing *NtHb1* exhibited higher levels of *NtHb1* transcript compared with that of the vector only-expressing control *Arabidopsis*, which had no *NtHb1* transcript. As expected, *NtHb1*-expressing *Arabidopsis* displayed higher tolerance to Cd than the control plants (Figure 1B,C). In contrast, the accumulation of Cd in *NtHb1*-*Arabidopsis* was lower than that in control plants (Figure 1D–F). Additionally, Cd was highly accumulated in the meristematic zone and stele (likely in the xylem).

### NO and ROS Are Less Induced by Cd in *NtHb1*-Expressing *Arabidopsis*

**FIGURE 2:**
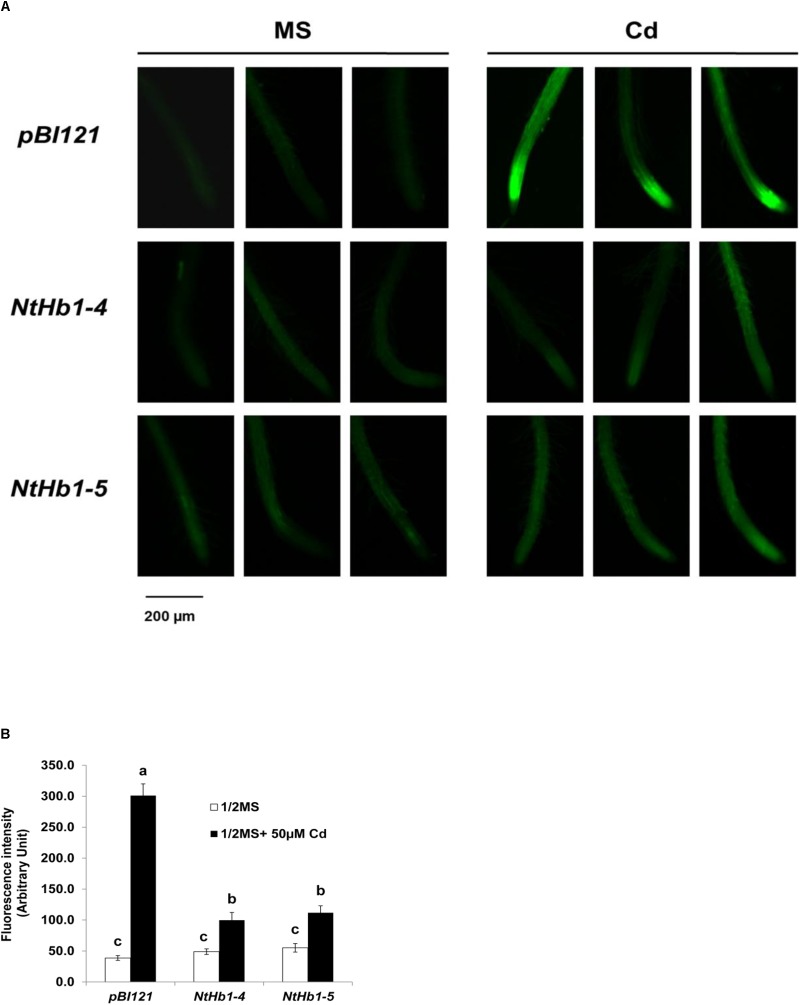
NO production in response to Cd in the control and *NtHb1*-expressing *Arabidopsis*. **(A)** NO production was visualized using DAF-2DA in the control (*pBI121*) and *NtHb1*-expressing *Arabidopsis* (*NtHb1*-4 and *NtHb1*-5), which were germinated and grown for 3 weeks on 1/2 MS agar media without (left) and with (right) 50 μM CdSO_4_. **(B)** The intensity of NO fluorescence (five plants from each line) was measured using Image J software. Each value corresponds to the means ± SE (*n* = 5). Different letters over the bars indicate significant differences according to Tukey’s multiple comparison test (*p* ≤ 0.05).

Because NO production is enhanced in response to Cd (Besson-Bard et al., 2009) and Hb scavenges NO in *Arabidopsis* (Dordas et al., 2003; Perazzolli et al., 2004; Hebelstrup et al., 2006), the NO level was examined in control and transgenic *Arabidopsis* germinated and grown with 50 μM CdSO_4_ for 21 days. As shown in Figure 2A,B, the NO level was highly enhanced (6.0-fold) by Cd in control *Arabidopsis* expressing the *pBI121* vector only. In contrast, the NO level was less increased (2-fold) by Cd in *NtHb1*-expressing *Arabidopsis*, indicating that Cd-induced NO accumulation was decreased by increased Hb in *NtHb1*-expressing *Arabidopsis*, leading to increased Cd tolerance. In addition, the increased accumulation of NO in the meristem and stele of Cd-treated plants (Figure 2A) corresponded to the pattern of Cd accumulation (Figure 1E).

**FIGURE 3:**
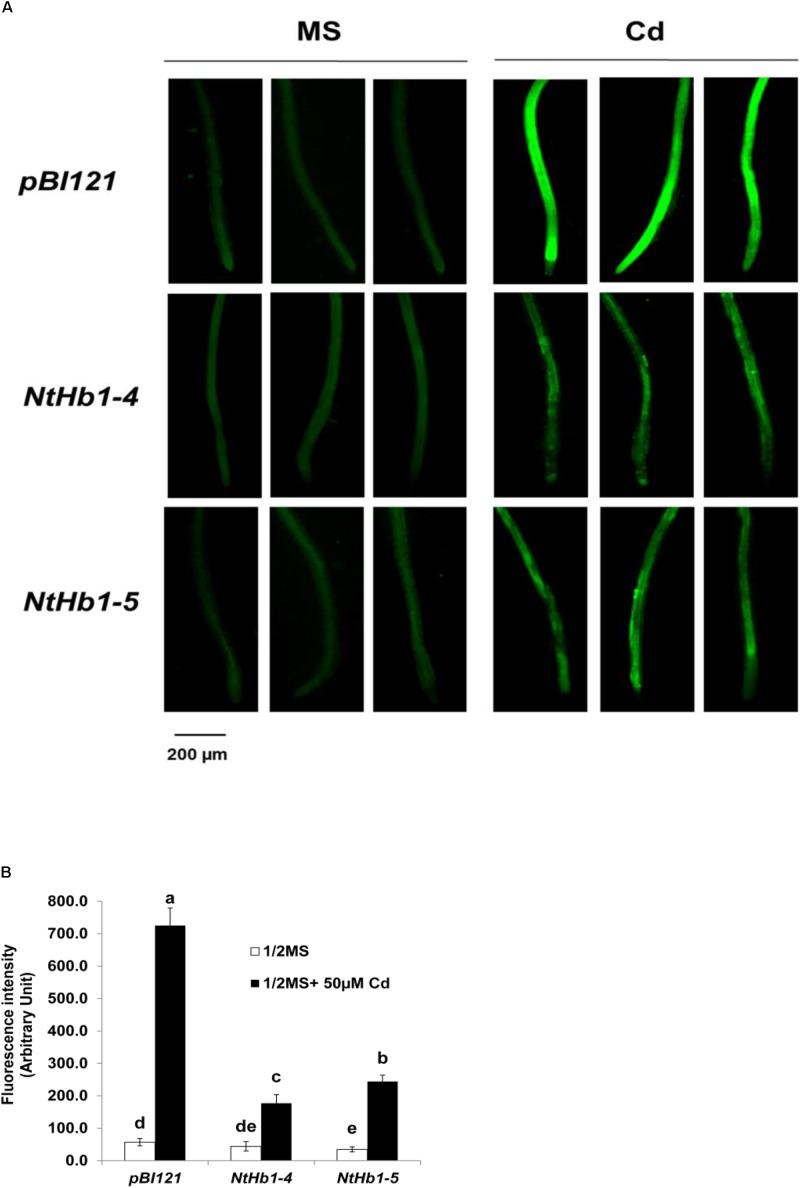
ROS levels in response to Cd in the control and *NtHb1*-*Arabidopsis*. **(A)** ROS staining with a fluorescent probe (CM-H_2_DCFDA). Plants were germinated and grown for 3 weeks on 1/2 MS agar media without and with 50 μM Cd. Experiments were performed 3 times, and 10 plants from each line were examined in each experiment. **(B)** Quantification of the ROS shown in **(A)**. The fluorescence signal was quantified using Image J software. Data are presented as the means of three independent experiments, and the error bars indicate SE. Different letters over the bars indicate significant differences according to Tukey’s multiple comparison test (*p* ≤ 0.05).

**FIGURE 4:**
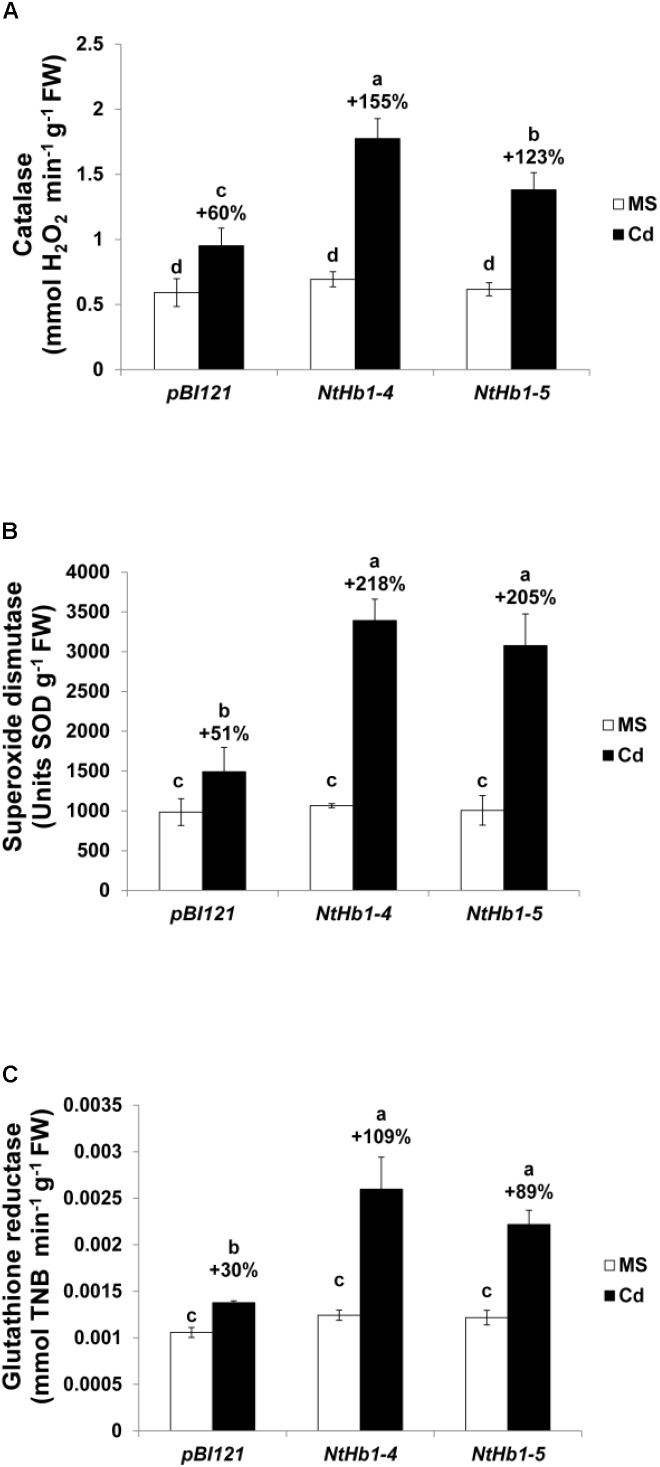
Activity of anti-oxidative enzymes in response to Cd in the control and *NtHb1*-expressing *Arabidopsis*. Activity of catalase **(A)**, superoxide dismutase **(B)**, and glutathione reductase **(C)** in control (*pBI121*) and *NtHb1 Arabidopsis*. Plants were germinated and grown for 3 weeks on 1/2 MS agar media without (white) and with (black) 50 μM Cd. Data correspond to the means of three independent experiments, and the error bars indicate SE. Different letters over the bars indicate significant differences according to Tukey’s multiple comparison test (*p* ≤ 0.05).

Because ROS are also generated by Cd in *Arabidopsis* (Bi et al., 2009), the ROS level was measured using a fluorescent probe (CM-H_2_DCFDA). As shown in Figure 3, ROS accumulation was greatly increased (14-fold) by Cd in control plants, while the ROS content was less enhanced (3∼4-fold) in *NtHb1*-expressing *Arabidopsis*. Interestingly, ROS evenly accumulated in the roots in response to Cd (Figure 3), while the Cd and NO levels were higher in the meristem and stele (Figure 2). To examine the involvement of anti-oxidative enzymes in decreasing ROS levels, the activities of CAT, SOD, and GR were measured in control and transgenic plants. As shown in Figure 4, all three enzymes were activated by Cd in both control and *NtHb1*-expressing *Arabidopsis* plants, but were highly induced in transgenic plants compared with levels in control plants.

### Expression of *CAXs* and *PDR8* Is Enhanced While That of *IRT1* Is Decreased in Response to Cd, Leading to Higher Cd Export in *NtHb1*-Expressing *Arabidopsis*

**FIGURE 5:**
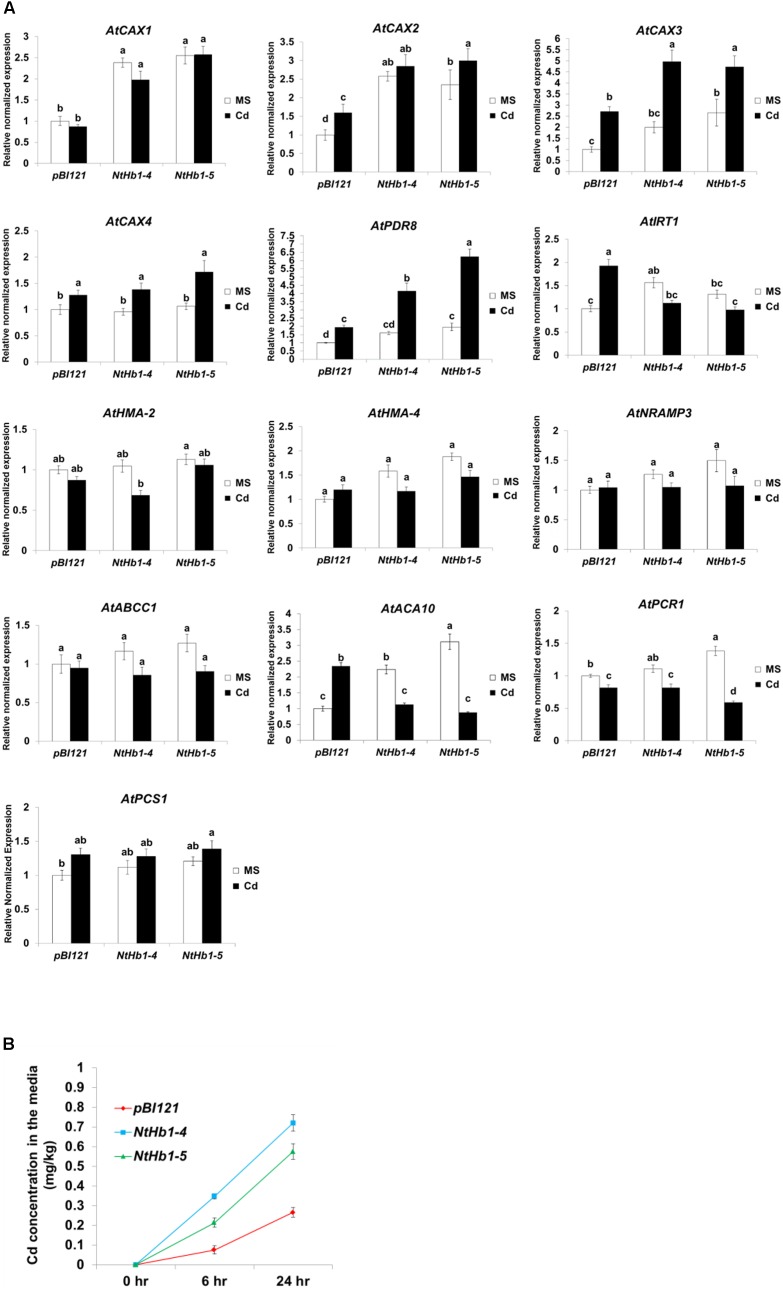
Expression of Cd transporter genes in response to Cd and Cd efflux in the control and *NtHb1*-*Arabidopsis*. **(A)** qRT-PCR results showing the transcript levels of various Cd transporter genes in transgenic *Arabidopsis*.Notably, *pBI121* refers to control plants containing the empty vector *pBI121*, and *NtHb1*-4 and *NtHb1*-5 refer to two different lines of *NtHb1*-expressing transgenic *Arabidopsis*. qRT-PCR was performed using total RNA isolated from plants that were germinated and grown for 3 weeks on 1/2 MS plates without or with 50 μM Cd. The relative transcript levels were assessed by normalization to actin transcript abundance as an endogenous control. **(B)** Cd efflux in plants. Control and transgenic plants were germinated and grown for 3 weeks on 1/2 MS agar plates, transferred to agar plates with 50 μM Cd, incubated for 24 h, and then placed in Cd-free liquid media for 6 h and 24 h. Subsequently, the Cd concentrations in liquid media were determined. All samples were run in triplicate (three biological repeats) for cDNA and each primer set, and the error bars indicate SE. Different letters over the bars indicate significant differences according to Tukey’s multiple comparison test (*p* ≤ 0.05).

To elucidate the mechanism by which Cd accumulation is decreased in *NtHb1*-*Arabidopsis*, qualitative reverse transcription–polymerase chain reaction (qRT-PCR) was performed to examine the expression levels of various Cd transporters, including *AtCAX1* (vacuolar transporter, *AT2G38170*), *AtCAX2* (vacuolar transporter, *AT3G13320*), *AtCAX3* (vacuolar transporter, *AT3G51860*), *AtCAX4* (vacuolar transporter, *AT5G01490*), *AtPDR8* (efflux transporter, *AT1G59870*), *AtIRT1* (uptake transporter, *AT4G19690*), *AtHMA2* (efflux transporter, *AT4G30110*), *AtHMA4* (xylem loading transporter, *AT2G19110*), *AtNRAMP3* (vacuolar transporter, *AT2G23150*), *AtABCC1* (vacuolar transporter, *AT1G30400*), *AtACA10* (putative uptake transporter, *OsACA6* homolog, *AT4g29900.1*), and *AtPCR1* (efflux transporter, *AT1G14880.1*). As shown in Figure 5A, the expression of *AtCAX3* and *AtPDR8* was increased by Cd in both control and *NtHb1*-expressing *Arabidopsis* plants, but transgenic plants showed greater increases and thus higher levels than those in control plants. In contrast, the expression of *AtIRT1* and *ACA10* was induced by Cd in control plants but reduced in *NtHb1*-*Arabidopsis*, resulting in a lower level of *IRT1* and *ACA10* in transgenic plants than in control plants. The expression of *CAX1* and *CAX2* in response to Cd was also higher in transgenic plants because the expression of these genes was already increased in transgenic plants in the absence of Cd. The expression of *AtHMA2*, *AtHMA4*, *AtNRAMP3*, *AtABCC1*, and *AtPCR1* did not show significant differences between control and transgenic plants in the absence and presence of Cd. Moreover, phytochelatins have been shown to play a significant role in Cd tolerance by sequestrating Cd in vacuoles (Pomponi et al., 2006; Brunetti et al., 2011). To examine whether phytochelatins are involved in Cd tolerance of *NtHb1*-expressing plants, we measured the expression level of *AtPCS1* (phytochelatin synthase, *AT5G44070*) in control and *NtHb1*-expressing plants. As shown in Figure 5A, the transcript level of *AtPCS1* did not change significantly in control and transgenic plants in the absence and presence of Cd, indicating that phytochelatins may not participate in enhancing the Cd tolerance observed in *NtHb1*-expressing transgenic plants. Furthermore, the Cd export rates of plants were examined. As shown in Figure 5B, *NtHb1*-expressing *Arabidopsis* showed higher Cd efflux rates than those of control plants, suggesting the involvement of CAX1∼3 and PDR8 in reducing Cd levels in transgenic plants.

Taken together, these results suggest that the increased expression of *CAX1∼3* and *PDR8* and the decreased expression of *IRT1* and *ACA10* are involved in reducing Cd accumulation by enhancing the Cd efflux and decreasing the Cd influx in *NtHb1*-expressing *Arabidopsis*.

### Expression of *CAX3*, *PDR8*, *IRT1*, and *ACA10* Is Regulated by NO and ROS in *Arabidopsis*

**FIGURE 6:**
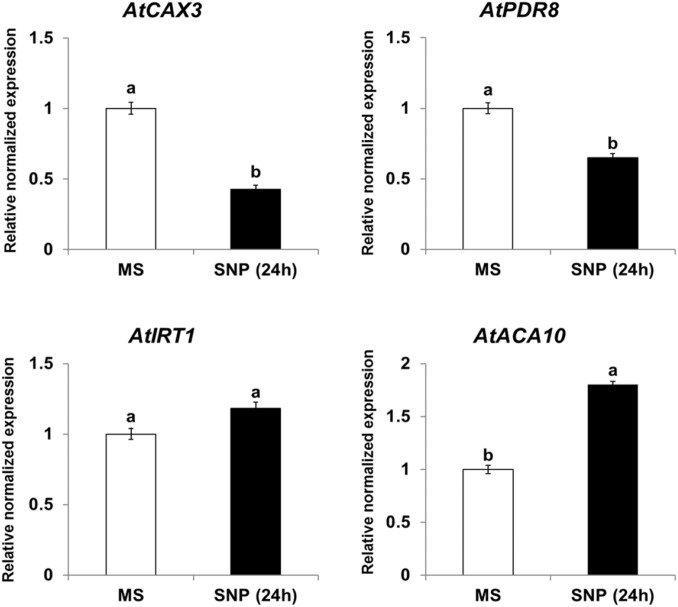
Short-term effect of SNP (NO donor) on the phenotype and gene expression of Cd transporters in Col-0 *Arabidopsis*. qRT-PCR results showing the transcript levels of various Cd-transporter genes in Col-0 *Arabidopsis* after short-term (24-h) exposure to SNP. qRT-PCR was performed using total RNA isolated from Col-0 plants, which were germinated and grown for 3 weeks on 1/2 MS agar plate and subsequently treated with 1/2 MS liquid (labeled as MS) and 500 μM SNP (labeled as SNP) for 24 h. All experiments were performed independently in triplicate, and the error bars indicate SE.

**FIGURE 7:**
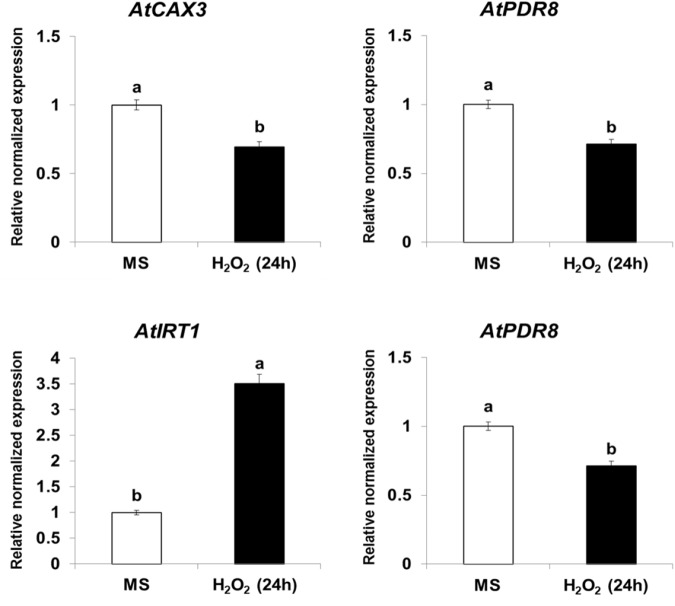
Short-term effect of H_2_O_2_ on the gene expression of Cd transporters in Col-0 *Arabidopsis*. qRT-PCR results showing the transcript levels of various Cd-transporter genes in Col-0 *Arabidopsis* after short-term (24-h) exposure to H_2_O_2_. qRT-PCR was performed using total RNA isolated from Col-0 plants, which were germinated and grown for 3 weeks on 1/2 MS agar plate and subsequently treated with 1/2 MS liquid (labeled as MS) and 100 μM H_2_O_2_ for 24 h.

Because *NtHb1*-*Arabidopsis* showed a lower level of NO, it is likely that the expression of Cd transporters was regulated by the decreased NO content. To examine this hypothesis, we treated Col-0 *Arabidopsis* with SNP (an NO donor) for 24 h and subsequently examined the expression of *CAX3*, *PDR8*, *IRT1*, and *ACA10* using qRT-PCR. As shown in Figure 6, when plants were germinated and grown for 3 weeks and thereafter treated with SNP for 24 h, the transcript levels of *CAX3* and *PDR8* were decreased, while those of *IRT1* and *ACA10* were increased, and these expression patterns were reversed in *NtHb1*-expressing *Arabidopsis* (Figure 5). In addition, the transcript levels of *CAX3* and *PDR8* were decreased, while those of *IRT1* and *ACA10* were increased in plants germinated and grown for 3 weeks and thereafter treated with H_2_O_2_ for 24 h (Figure 7). These data indicate that the enhanced expression of *CAX3* and *PDR8* and decreased expression level of *IRT1* and *ACA10* in *NtHb1-Arabidopsis* are attributed to the reduced levels of NO and ROS.

### *AtHb1* Expression Is Enhanced Greatly in the Roots and Slightly in the Shoots in Response to Cd

**FIGURE 8:**
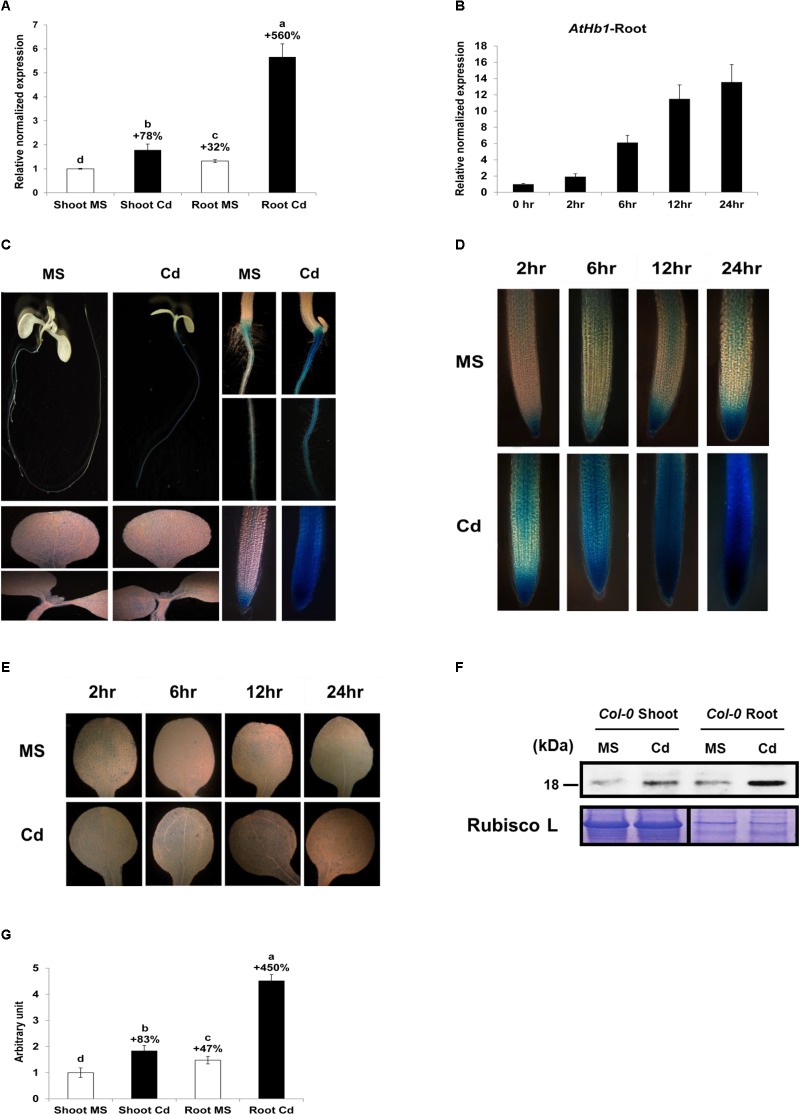
Long-term and short-term effect of Cd on *AtHb1* expression in Col-0 *Arabidopsis*. **(A)** Long-term (3-week) effect of Cd on the expression level (qRT-PCR) of *AtHb1* in *Arabidopsis*. Plants were germinated and grown for 3 weeks on 1/2 MS agar media without and with 50 μM Cd. The values over the bars indicate the increment compared with the shoot MS. **(B)** Short-term (24-h) effect of Cd on *AtHb1* expression. Plants were germinated and grown for 2 weeks on 1/2 MS agar media and then treated with 50 μM Cd for 2, 6, 12, and 24 h. **(C)** GUS staining of *pAtHB1::GUS* plants after long-term (7-day) exposure to Cd. Plants were germinated and grown for 7 days on 1/2 MS agar media with 50 μM Cd and stained. **(D,E)** GUS staining of *pAtHB1::GUS* plants after short-term (24-h) exposure to Cd. Plants were germinated and grown for 7 days on 1/2 MS agar media and then treated with Cd for 2, 6, 12, and 24 h, followed by staining. **(F)** Western blot of *AtHb1* protein in Col-0 *Arabidopsis*. Plants were germinated and grown on 1/2 MS and 1/2 MS with 50 μM Cd for 3 weeks, and protein was extracted from the roots and shoots. Rubisco L, used as a loading control for shoots, refers to the large subunit of RuBP carboxylase/oxygenase. **(G)** Quantification of the *AtHb1* protein shown in **(F)** by densitometry. The values over the bars indicate the increment compared with the shoot MS. Data correspond to the means of three independent experiments. Data of *pAtHB1::GUS* plants were produced from one transgenic line used as the representative of three transgenic lines.

To examine whether *AtHb1* is naturally involved in decreasing the NO level, which is induced by Cd in plants, the transcript level of *AtHb1* in response to Cd was measured (Figure 8). The expression of *AtHb1* was increased by 78% in the shoots and 428% in the roots in Col-0 *Arabidopsis* germinated and grown for 3 weeks on Cd media (Figure 8A). The *AtHb1* expression level in the roots was 32 and 317% higher, respectively, than that in the shoots in the absence and presence of Cd. The *AtHb1* transcript level was elevated by up to 14-fold in the roots within 24 h after Cd treatment (Figure 8B). Furthermore, the *AtHb1* protein level was increased by 83% in the shoots and 305% in the roots in response to Cd, while the *AtHb1* level was 47 and 246% higher, respectively, in the roots than in the shoots in the absence and presence of Cd (Figure 8F,G). In addition, transgenic *Arabidopsis* expressing the *pAtHb1*-*GUS* construct was generated to study the tissue localization of *AtHb1*. As shown in Figure 8C,E, *AtHb1* was highly expressed in the roots but slightly expressed in cauline leaves. In the absence of Cd, *AtHb1* was highly expressed in the root tips (meristems) and slightly expressed around the stele in the upper part of the roots. In contrast, *AtHb1* expression was highly induced by Cd in every part of the roots, with the highest level of expression in the meristem, which corresponds to the NO pattern (Figure 2A, 8D), but *AtHb1* induction was not clear in the shoots (Figure 8C,E). Regarding the 78% induction of the *AtHb1* transcript levels by Cd in the shoots, the non-induction of GUS by Cd in *pAtHb1*-*GUS* plants may be ascribed to the shorter Cd treatment (7 days) compared with the 3-week Cd treatment in the *AtHb1* transcript measurement, while GUS expression in the roots was enhanced by the 7-day Cd treatment. The time-dependent slight increase in *Hb1* expression without Cd challenge (Figure 8D) was likely due to a buffer-induced hypoxia condition. These data suggest that Hb1 is naturally involved in removing NO, which is induced by Cd in the roots. *AtHb1* expression was observed in cauline leaf tips and rosette leaf hydathodes in *Arabidopsis* Col-0 (Hebelstrup et al., 2006). Furthermore, *AtHb1* expression was also observed in the shoot apical meristem of 16-day-old *Arabidopsis* plants (Hebelstrup and Jensen, 2008). By contrast, *AtHb1* was only expressed in the roots of the plant (C-24), and it was absent in the shoots (Hunt et al., 2001).

## Discussion

Transgenic *Arabidopsis* expressing the hemoglobin gene *NtHb1* showed an enhancement in Cd tolerance and reductions in Cd, NO, and ROS levels. The increase in Cd tolerance could be ascribed to the decreased Cd accumulation, as well as the reduction in oxidative stress, including NO and ROS. The reduction in the Cd level might be due to the enhanced expression of *CAX3* and *PDR8* and reduced expression of *IRT1* and *ACA10*, which was attributed to the diminished levels of NO and ROS.

### NO Accumulation Is Increased by Cd and Inhibited by Hemoglobin in *Arabidopsis*

Nitric oxide levels were enhanced by Cd in both control and *NtHb1*-expressing *Arabidopsis*, but NO was less increased (103%) in transgenic plants than in control plants (679%), resulting in lower levels of NO in transgenic plants (Figure 2). This finding suggests that the enhanced Hb1 diminishes the NO level, likely by scavenging more NO in *NtHb1*-expressing *Arabidopsis*.

The NO level was enhanced by Cd treatment in diverse plant species, including wheat (Groppa et al., 2008), *Arabidopsis* (Besson-Bard et al., 2009; De Michele et al., 2009; Zhu et al., 2012; Ye et al., 2013), white poplar (Balestrazzi et al., 2011), barley (Valentovicova et al., 2010), tobacco (Ma et al., 2010; Kulik et al., 2012; Lee and Hwang, 2015), pea (Lehotai et al., 2011), yellow lupine (Arasimowicz-Jelonek et al., 2012), Indian mustard (Verma et al., 2013), and soybean (Perez-Chaca et al., 2014). In contrast, some studies reported that NO content was decreased by Cd (Rodriguez-Serrano et al., 2009; Xiong et al., 2009; Ortega-Galisteo et al., 2012; Gupta et al., 2017).

### Decreased NO and Cd Levels Reduce the ROS Level, Contributing to Cd Tolerance

In addition to the lower accumulation of NO in response to Cd, *NtHb1*-expressing *Arabidopsis* also had a lower ROS level (Figure 3). The lower levels of NO and ROS contribute to Cd tolerance higher in transgenic plants than that in control plants. Cd toxicity is mediated by NO by increasing ROS (De Michele et al., 2009; Arasimowicz-Jelonek et al., 2012; Kulik et al., 2012), RNS (Arasimowicz-Jelonek et al., 2012), and lipid peroxidation (Groppa et al., 2008; Zhu et al., 2012) as well as by inhibiting the anti-oxidative enzymes CAT and ascorbate peroxidase (De Michele et al., 2009). These reports suggest that Cd toxicity may not be enhanced if NO is not elevated in response to Cd. Consistently, in transgenic *Arabidopsis* expressing *NtHb1*, the NO level was less elevated in response to Cd, while Cd tolerance was more promoted than it was in control plants (Figure 1B,C, 2). Therefore, we concluded that the enhancement of Cd tolerance in *NtHb1*-*Arabidopsis* is attributed to the reduced accumulation of NO, which is responsible for Cd toxicity by promoting oxidative stress.

Furthermore, the lower ROS content in transgenic plants suggests that the ROS level is also decreased by Hb and that NO functions as ROS or produces ROS in *Arabidopsis*. In support of this result, it was reported that Hb was involved in decreasing ROS in *Arabidopsis* (Yang et al., 2005), and NO increased the ROS level (De Michele et al., 2009; Arasimowicz-Jelonek et al., 2012; Kulik et al., 2012). In addition, the highly enhanced activities of the anti-oxidative enzymes CAT, SOD, and GR by Cd in transgenic plants are responsible for the smaller increase of ROS (Figure 4). The higher activities of anti-oxidative enzymes in transgenic plants may be ascribed to the lower levels of NO and Cd. Interestingly, ROS accumulated evenly in roots in response to Cd (Figure 3), whereas the NO level was higher in the meristem and stele, indicating that the pattern of ROS production/accumulation is different from that of NO synthesis/accumulation in response to Cd in *Arabidopsis*.

### Decreased NO and ROS Levels Reduce Cd Accumulation by Enhancing Cd Efflux via Regulation of the Expression of Cd Transporters

Cd-tolerant *NtHb1*-expressing *Arabidopsis* displayed lower levels of Cd and NO than those of control plants (Figure 1D–F, 2). This result is supported by reports that NO mediates Cd toxicity by enhancing Cd levels (Ma et al., 2010; Arasimowicz-Jelonek et al., 2012; Chmielowska-Bąk and Deckert, 2013; Han et al., 2014). Among the putative Cd transporters examined, the expression of *AtCAX1∼3* (vacuolar transporter) and *AtPDR8* (efflux transporter) was higher in *NtHb1*-*Arabidopsis* than in control plants in the presence of Cd, while the *AtIRT1* (uptake transporter) and *AtACA10* (putative uptake transporter) transcript levels were lower. This finding suggests that the altered expressions of these transporters are involved in decreasing Cd accumulation in *NtHb1*-*Arabidopsis*. This is supported by the enhanced Cd efflux in transgenic plants (Figure 5B). *IRT1* expression is induced by Cd exposure in tobacco (Yoshihara et al., 2006), and the over-expression of *IRT1* reduces Cd tolerance in *Arabidopsis* and rice, which is accompanied by increased accumulation of Cd (Connolly et al., 2002; Lee and An, 2009). The proposed roles for PDR8 and IRT1 in *NtHb1*-expressing *Arabidopsis* are supported by previous reports that PDR8 functions in pumping out Cd from the cytoplasm (Kim et al., 2007) and IRT1 plays a role in Cd absorption (Korshunova et al., 1999; Connolly et al., 2002). Like *IRT1*, *ACA10* expression was induced by Cd in control plants but was reduced in transgenic plants, leading to a lower level than that in control plants (Figure 5A). In support of our result, the transcript level of *OsACA6* (homologous to *Arabidopsis ACA10*) was increased by Cd treatment in rice, and tobacco expressing *OsACA6* exhibited higher Cd tolerance and accumulation in the roots (Shukla et al., 2014). It has been reported that Cd uptake can be mediated by the transporters involved in uptake and transport of other divalent cations, such as Ca^2+^, Fe^2+^, and Mn^2+^ (Vert et al., 2002; Sasaki et al., 2012; Chen et al., 2018). However, it was not elucidated how OsACA6 expression enhanced Cd levels in roots.

In contrast, it is not clear how CAXs are involved in reducing the Cd levels in transgenic plants because CAXs have been reported as tonoplast transporters that sequester Ca, Cd, and Mg into vacuoles in *Arabidopsis* (Pittman and Hirschi, 2016). Therefore, enhanced CAXs may indirectly participate in decreasing Cd accumulation in transgenic *Arabidopsis* through modulation of the cytosol Ca concentration. Furthermore, other studies suggest that CAXs may not contribute to reducing Cd accumulation in plants. *AtCAX1*-expressing petunia showed higher Cd tolerance and accumulation (Wu et al., 2011), and the ectopic expression of *Arabidopsis CAX2* and *CAX4* improved Cd tolerance along with higher Cd accumulation (Korenkov et al., 2007). In addition, CAXs can promote Cd tolerance by regulating ROS signaling and accumulation in plants. Knockout *Arabidopsis* of *CAX1* showed less tolerance to Cd at low concentration of Ca and a higher ROS level compared with the WT plant (Baliardini et al., 2015). Very recently, *CAX1* has been shown to increase the Cd tolerance and diminish ROS accumulation under Cd stress in *Arabidopsis halleri* (Ahmadi et al., 2018). Therefore, it is very possible that the higher expression level of *CAXs* in *NtHb1*-*Arabidopsis* also lowered ROS accumulation compared with the control plants, resulting in a higher Cd tolerance.

Sodium nitroprusside (NO donor) treatment experiments (Figure 6) demonstrated that expression of *CAX3* and *PDR8* was decreased by NO, but *IRT1* expression was enhanced, emphasizing the correctness of the reversed expression pattern in *NtHb1*-expressing *Arabidopsis*. Expression of *IRT1* is enhanced by NO, which leads to an increase in Cd uptake (Besson-Bard et al., 2009; Luo et al., 2012; Zhu et al., 2012). However, the repression of *CAX3* and *PDR8* expression, as well as induction of *ACA10* expression, by NO was first reported in this research. Regarding the increased expression of *CAX1∼3*, *PDR8*, and *ACA10* in *NtHb1*-*Arabidopsis* in the absence of Cd (Figure 5), the higher level of Hb1 in transgenic plants is likely involved in enhancing the expression of *CAX1∼3*, *PDR8*, and *ACA10* by decreasing NO, even without a Cd challenge. In summary, the reduced accumulation of Cd in *NtHb1*-*Arabidopsis* was attributed to the enhanced expression of *CAX1∼3* and *PDR8*, and to the reduced expression of *IRT1* and *ACA10*, which was caused by the decreased level of NO.

It seems that ROS, in addition to NO, is able to regulate the expression of Cd transporters. In response to H_2_O_2_ treatment, the transcript levels of *CAX3* and *PDR8* were decreased, while those of *IRT1* and *ACA10* were increased (Figure 7). *NtCAX3* expression was increased and *NtIRT1* expression was reduced in *NtUBC1*-expressing tobacco, which accumulated a lower level of ROS under Cd stress conditions (Bahmani et al., 2017). Moreover, *NRAMP3* and *NRAMP4* were up-regulated by H_2_O_2_ treatment (Molins et al., 2013), and *ABCC3*, *ABCC4*, and *ABCC6* expression was also enhanced in wheat plants exposed to H_2_O_2_ (Bhati et al., 2015). The next step is to elucidate how NO or ROS regulates gene expression of Cd transporters.

**FIGURE 9:**
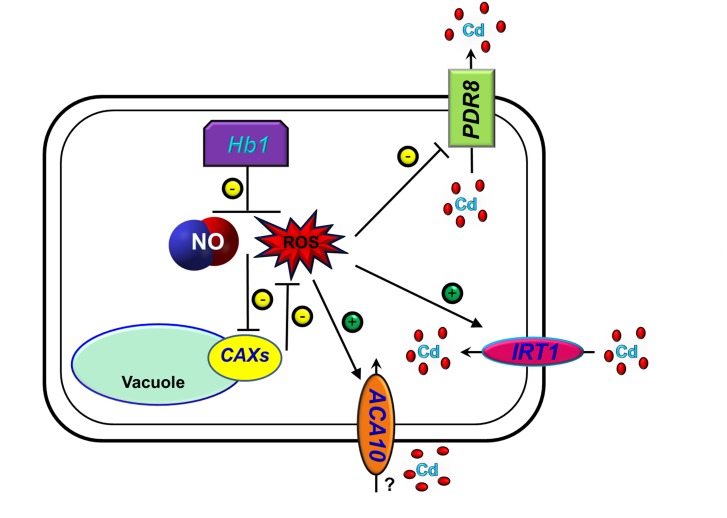
A proposed model for the function of Hb1 in response to cadmium. The arrows show positive regulation, whereas the blunt lines show negative regulation.

In summary, we propose a model (Figure 9) showing that the over-expression of *NtHb1* reduces NO and ROS levels, by which Cd efflux is enhanced and Cd influx is decreased.

## Conclusion

The expression of *NtHb1* in *Arabidopsis* reduces Cd levels and increases Cd tolerance by inhibiting expression of *IRT1* and *ACA10* and enhancing expression of *PDR8* and *CAXs* via decreased NO and ROS (H_2_O_2_) levels. In addition, *AtHb1* expression is greatly enhanced in the roots and slightly enhanced in the shoots in response to Cd.

## Author Contributions

SH acquired a research fund, designed and supervised whole research, and wrote the article with contributions of all authors. RB performed most experiments. DK and JN performed the minor parts of experiments.

## Conflict of Interest Statement

The authors declare that the research was conducted in the absence of any commercial or financial relationships that could be construed as a potential conflict of interest.

## Footnotes

1 www.thermofisher.com/order/catalog/product/C6827

## Abbreviations

ANOVA: analysis of variance
CaCl_2_: calcium chloride
CAT: catalase
CM-H2DCFDA: 5-(and-6)-chloromethyl-2′,7′-dichlorodihydrofluorescein diacetate
cPTIO: 2-4-carboxyphenyl-4,4,5,5-tetramethylimidazoline-1-oxyl-3-oxid
DAF-2DA: 4,5-diaminofluorescein
DMSO: Dimethyl sulfoxide
DTNB: 2-nitrobenzoic acid
DTT: dithiothreitol
EDTA: ethylene diamine tetraacetic acid
EGTA: ethylene glycol tetraacetic acid
GR: glutathione reductase
GSSG: glutathione disulphide
H_2_O_2_: hydrogen peroxide
ICP: inductively coupled plasma, MS, Murashige and Skoog
NBT: nitroblue tetrazolium
NO: nitric oxide
qRT-PCR: quantitative real-time PCR
RNS: reactive Nitrogen Species
ROS: reactive oxygen species
SNP: sodium nitroprusside
SOD: superoxide dismutase
TNB: 2-nitro-5-thiobenzoate
WT: Wild type.
