# Supplementary material for: Expression of the Tobacco Non-symbiotic Class 1 Hemoglobin Gene Hb1 Reduces Cadmium Levels by Modulating Cd Transporter Expression Through Decreasing Nitric Oxide and ROS Level in Arabidopsis
Source: Front Plant Sci. 2019 Feb 22;10:201. doi: 10.3389/fpls.2019.00201 (PMC6396062; doi:10.3389/fpls.2019.00201)
Supplement: Supplementary file 1 [file Table_1.DOCX]

Supplementary Material

Expression of the *tobacco* non-symbiotic class 1 hemoglobin gene *Hb1* reduces cadmium levels by modulating Cd transporter expression through decreasing nitric oxide and ROS level in *Arabidopsis*

**Ramin Bahmani^1,2,3^, DongGwan Kim^1,2,3^, JongDuk Na^1^ and Seongbin Hwang^1,2,3*^**

Dept. of Molecular Biology^1^, Dept. of Bioindustry and Bioresource Engineering^2^, and Plant Engineering Research Institute^3^, Sejong University, Seoul, 143-747, KOREA

**^*^Correspondence:** Corresponding Author: [sbhwang@sejong.ac.kr](mailto:sbhwang@sejong.ac.kr)

# Supplementary Tables

**Supplemental Table S1.** Sequences of primers used for cloning and gene expression (q-RT PCR) analysis.

**1-1 Supplemental Table S1.** Sequences of primers used for cloning and gene expression (q-RT PCR) analysis.

| **Gene name** | **Locus** | **Primer Sequences** |
| --- | --- | --- |
| *AtActin2* | AT3G18780 | F: 5′-GCAGAGCGGGAAATTGTAAG-3′ |
|  |  | R: 5′-TTCTCGATGGAAGAGCTGGT-3′ |
| *AtCAX1* | AT2G22475 | F: 5′-TGCTTGGTAGATGGGGAAAG-3′ |
|  |  | R: 5′-ATTCTTCCCATTGCAGCATC-3′ |
| *AtCAX2* | AT5G24520 | F: 5′-CGAGCATCCTTATCCTCCAA-3′ |
|  |  | R: 5′-GCTCGACGGTTGATGAATCT-3′ |
| *AtCAX3* | AT1G79840 | F: 5′-ACGTGGGACAATGGAAAGAG-3′ |
|  |  | R: 5′-AGCTGCATCTCTCCGAACAT-3′ |
| *AtCAX4* | AT1G66470 | F: 5′-CCTAAATCCGCTGGAAACAA-3′ |
|  |  | R: 5′-TTGTTGGCTTAGGCTTGGTC-3′ |
| *AtHMA2* | AT3G23050 | F: 5′-GAACTTTGGTGGAGGAGCAG-3′ |
|  |  | R: 5′-GGCCAATGCATCAGAAAGAT-3′ |
| *AtHMA4* | AT2G38120 | F: 5′-CCACTCCAACGCTTTCTCTC-3′ |
|  |  | R: 5′-TGTCATGCATCCCAATCACT-3′ |
| *AtNRAMP3* | AT1G05180 | F: 5′-AACGGCTCAGTACTCCCAGA-3′ |
|  |  | R: 5′-CGGAACAAACTGCTTTGTGA-3′ |
| *AtPDR8* | AT1G66340 | F: 5′-GTACACCTTGGGTGCGAAGT-3′ |
|  |  | R: 5′-CGTGAATACGGAGAGCGATT-3′ |
| *AtABCC1* | AT5G03730 | F: 5′-CGGTCACTCAACCTCCAAAT-3′ |
|  |  | R: 5′-ACTCAGGCGACGTCTCTCAT-3′ |
| *AtIRT1* | AT4G19690 | F: 5′-AGCTTTGATCACGGTTGGAC-3′ |
|  |  | R: 5′-AGAAGAGCCGCGATTAAACA-3′ |
| *AtACA10* | AT4G29900.1 | F: 5′-GCACGCCCAAGCAATAAGAG-3′  R: 5′-TCTGCTCCTGTCCGATACCA-3′ |
| *AtPCR1* | AT1G14880.1 | F: 5′-GGTCCACAGGCTTCTGTGAT-3′  R: 5′-CCGCACAACACGATTTGGAT-3′ |
| *AtPCS1* | AT5G44070 | F: 5′-TTGTTGTCGGGAAACTTGCG-3′  R: 5′-TTCATTCCCATCACGCACCA-3′ |
| *NtHB1*  (cloning) | AII99810.1 | F- 5’-GCGTCTAGAATGAGTAGCTTTACAGAAGA-3’  R- 5’-ATAGGATCCCTACTTCATCTCGGTCTTGA-3’ |
| *AtHB1*(promoter)  (cloning) | AT2G16060 | F- 5’-TCTAGATTAAGGAGTACAAAGGGAA-3’  R- 5’-AAGCTTTTGTGTTACCGAAGAGACAGT-3’ |
